# Histopathological, immunohistochemical and biochemical alterations in liver tissue after fungicide-mancozeb exposures in Wistar albino rats

**DOI:** 10.1590/acb370404

**Published:** 2022-06-27

**Authors:** Ertuğrul Gök, Engin Deveci

**Affiliations:** 1 Assistant professor. Dicle University – Faculty of Medicine – Department of Forensic Medicine – Diyarbakir, Turkey.; 2 PhD. Dicle University – Faculty of Medicine – Department of Histology and Embryology – Diyarbakir, Turkey.

**Keywords:** Immunohistochemistry, Toxicity, Liver

## Abstract

**Purpose:**

To evaluate the histopathological, immunohistochemical, and biochemical effects of liver changes after mancozeb administration.

**Methods:**

Rats were divided into groups–the control group (n=7) and the mancozeb group (n=7)–, given 500 mg/kg mancozeb dissolved in corn oil daily for four weeks by an orogastric tube. Caspase-3 and tumor necrosis factor-alpha (TNF-α) primary antibodies were used for immunohistochemical analysis.

**Results:**

Serum aspartate aminotransferase (AST) and alanine aminotransferase (ALT) values of the mancozeb group increased significantly than ones of the control group. Venous dilatation, inflammation, hepatocyte degeneration, TNF-α, and caspase-3 expression scores increased significantly in the mancozeb group. In the mancozeb group, intensive caspase-3 expression was observed in hepatocyte cells around the central vein in the center of the liver lobule, and there was an increase in TNF-α expression in the inflammatory cells around the enlarged central vein and Kupffer cells and apoptotic hepatocyte cells.

**Conclusions:**

Subacute mancozeb exposure in rats leads to elevated toxicity with impaired liver function, increased inflammation in tissue and increased apoptosis due to cellular damage in the liver, and decreased liver regeneration ability due to congestion and degeneration of blood vessels.

## Introduction

Intoxication is one of the fields of study in forensic medicine, and pesticides are agents leading to intoxication. Throughout the world, pesticides are widely being used in agriculture for food production. As a result of this extensive pesticide use, humans are regularly exposed to pesticides and residues of them. Food and drinking water intake are the primary sources of contamination of the general population through persistent accumulative pesticide residues in the environment^1^^,^ 2.

One of these pesticides is mancozeb, a fungicide. It is a commonly used broad-spectrum fungicide that takes under control a significant number of fungal diseases in agriculture and horticulture^3^^-^ 5. It is a manganese/zinc ethylene-bis-dithiocarbamate fungicide, and, through reacting with sulfhydryl groups of amino acids and enzymes, it inactivates these amino acids and enzymes in fungal cells^2^. As a result of this inactivation, disruptions occur in the lipid metabolism, respiration, and production of adenosine triphosphate^6^.

Mancozeb exposure is frequent among chemical industry and agricultural workers after inhalation of dust or sprays, dermal contact, or accidental/incidental ingestion^2^. The body metabolizes the mancozeb quickly, and animal studies show low-acute toxicity. However, experiments on rodents presented that mancozeb and its metabolite, ethylene thiourea (ETU), can cross the placental barrier and disrupt reproductive performance, lead DNA damage, and also initiate fetal cell tumors^2^^,^ 7 ^-^ 9. Mancozeb is a multipotent carcinogen with long-term exposure in laboratory animals^10^. In rodents, it has the potential to cause thyroid and hepatic cancers^11^. Besides, as a result of inducing a reduction in T4 levels in female rats, which may harm the developing brain, it is known as an endocrine system disruptor associated with hypothyroidism and hyperthyroidism^12^^,^ 13.

Experiments conducted in vitro have shown that mitochondrial enzymes are the main targets of the fungicides^14^^,^ 15. Furthermore, the formation reactive oxygen species are stimulated by fungicides in rat fibroblasts^16^. Humans exposed to mancozeb exhibited a series of health problems such as neurotoxic effects and Parkinson-like symptoms and sensitivity in children and women, such as thyroid hormone disruption and dysregulation in fetal brain development-neural tube defects^13^^,^ 17 ^,^ 18. Neoplastic changes are seen *in vitro* and mouse skin *in vivo* 19. Mancozeb causes alterations in various biochemical enzymes^20^. These enzymatic changes may be associated with pathomorphological changes in the liver, kidney, and brain. Hepatocellular damage such as necrosis is thought to be related to changes in tissue and serum enzymes such as alkaline phosphatase. Administration of mancozeb may cause a decreased serum cholinesterase activity and lead to slow enzyme synthesis in the damaged liver, and also inhibition of microsomal oxygenases produce liver damage. Subchronic mancozeb treatment leads to liver toxicity in female Wistar rats^21^. Cell death in the liver is a result of apoptosis or necrosis.

To isolate damaged or infected cells to preserve tissue integrity while at the same time preventing inflammation and damaging other formations, this process is followed by apoptosis. Both increased mitochondrial lysosomal permeability and reactive oxygen species (ROS) formation mediate death receptor-induced apoptosis; thus, hepatocytes promote apoptosis in response to toxic substances^22^^,^ 23. Hepatic hypoxia has been reported to cause caspase-3 activations in hepatocytes^24^. Tumour necrosis factor-α is a multifunctional proinflammatory cytokine that controls a wide range of metabolism, insulin sensitivity, inflammation, cellular apoptosis, coagulation, tumor growth and invasion, and vascular functions^25^^,^ 26. The purpose of this investigation was to show the histopathological, immunohistochemical, and biochemical effects in the liver after mancozeb administration.

## Methods

### Experimental animals and study design

The research was carried out in line with the Guide for the Care and Use of Laboratory Animals published by the U.S. National Institutes of Health in 2011. All procedures performed in this experiment were approved by the Ethics Committee for the Treatment of Experimental Animals (Dicle University, Turkey, protocol number: 2021/03). The rats were provided by Dicle University, Department of Medical Sciences Research and Application Center.

Animals had an acclimation period of 72 h before use in the study. Two groups were formed from 14 young adults (60 days) female Wistar albino rats, weighed 180-210 g, and were kept under standard laboratory conditions; 12/12 h light/dark period, with 50–70% humidity, at 23±2°C in standard steel cages in the room temperature. Rats were fed as *ad libitum* with standard rat pellets and water. Rats showed behavioral changes after the mancozeb administration for four weeks. Malnutrition and decrease in food and water intake were observed.

Mancozeb group rats (n=7) were given 500 mg/kg mancozeb dissolved in corn oil daily for four weeks, orally given by an orogastric tube^27^^,^ 28.

Control animals (n=7) were given the same daily quantity of corn oil in the same manner without mancozeb as the treated animals.

Next, under ether anesthesia, the animals were euthanized by cardiac exsanguination. Blood samples were collected by cardiac puncture.

### Biochemical analyses

Blood samples were placed in the tubes, and then centrifugation was done. Serum aspartate aminotransferase (AST) and alanine aminotransferase (ALT) values were determined at U/L using Abbott Architect c16,000 Autoanalyzer.

### Histopathological method

The samples were placed in 10% formaldehyde and dehydrated in 70–100% ethanol series. Next, for paraffin inclusion, they were placed in paraffin baths at 58°C. A rotary microtome was used to prepare 4–6 µm sections from paraffin blocks, which were later stained with hematoxylin-eosin.

### Immunohistochemistry method

First, for 5 min and then for 4 min, the antigen retrieval process was conducted to the sections that remained in a citrate buffer solution (pH=6) boiled in a microwave oven at 700 W (Bosch^^®^^). Later, they were cooled until room temperature for 20 min and washed twice for 5 min in distilled water. For 10 min, endogenous peroxidase activity was blocked in 0.1% hydrogen peroxide [K-40677109,64271 hydrogen peroxide (H_2_O_2_), Merck, Germany] (3-mL 30% hydrogen peroxide (H_2_O_2_) + 27-mL methanol). Before applying primary antibodies, caspase-3 mouse monoclonal, 1/100, and tumor necrosis factor-alpha mouse monoclonal, 1/100 for overnight, ultra V block (Histostain-Plus kit, Invitrogen, Carlsbad, CA, United States of America) was performed for 8 min. Following this, the secondary antibody (Histostain-Plus kit, Invitrogen, Carlsbad, CA, United States of America) was applied for 20 min. Next, for 25 min, the slides were applied streptavidin-peroxidase. For chromogen, diaminobenzidine (DAB, Invitrogen, Carlsbad, CA, United States of America, lot: HD36221) was used.

With the exclusion of primary antibodies, control slides were prepared, as already mentioned. Following the procedures of counterstaining with hematoxylin (product number: HHS32 SIGMA, haematoxylin solution, Harris Modified, Sigma-Aldrich, United States of America), and washing with tap water for 3 min and with distilled water for 2 × 3 min, Entellan^®^ (lot: 107961, Sigma-Aldrich, United States of America) was used to mount the slides.

### Statistical analysis

All statistical analyses were performed using Statistical Package for the Social Sciences (SPSS) version 24.0 (IBM, United States of America) software. The biochemical data of the control and the mancozeb groups were compared. Kolmogorov-Smirnov test was used as a normality test. As a result of the analysis, it was observed that the data were not distributed normally. For this reason, statistical evaluation was conducted with a non-parametric Mann-Whitney’s U test, and p<0.05 was considered statistically significant. Results were expressed as median (IQR), and boxplot graphs were used for demonstration.

## Results

It was found that there were significant differences between the control and the mancozeb groups in terms of AST and ALT levels, venous dilatation, inflammation, hepatocyte degeneration, TNF-α, and caspase-3 expression scores (Table 1). The animals were analyzed statistically by measuring their weights before and after the experiment. Weight loss due to mancozeb administration was significant (p<0.001).

**Table 1:** Effect of mancozeb on liver function tests and liver tissue. Data represent the group median (25-75%) values. Statistical significance is present between the control group and the mancozeb group in terms of all parameters (AST, ALT, and TNF-α).

Parameter	Control (n=7) (Median%-%)	Mancozeb (n=7) (Median%-%)	p-value
AST (U/L)	121.64 (119.22-122.44) (25-75)	170.25 (168.24-171.88) (25-75)	<0.01*
ALT (U/L)	111.46 (109.56-112.38)	141.77 (140.12-142.44)	<0.01*
Venous dilatation	0.00 (0.00-1.00)	2.00 (2.00-3.00)	<0.01*
Inflammation	1.00 (0.00-1.00)	3.00 (3.00-4.00)	<0.01*
Hepatocyte degeneration	1.00 (0.00-1.00)	3.00 (3.00-4.00)	<0.01*
TNF-α expression	1.00 (1.00-1.00)	3.00 (3.00-4.00)	<0.01*
Caspase-3 expression	1.00 (1.00- 1.00)	3.00 (3.00-4.00)	<0.01*

AST: aspartate aminotransferase; ALT: alanine aminotransferase; TNF-α: tumor necrosis factor-alpha.

**Figure 1:**
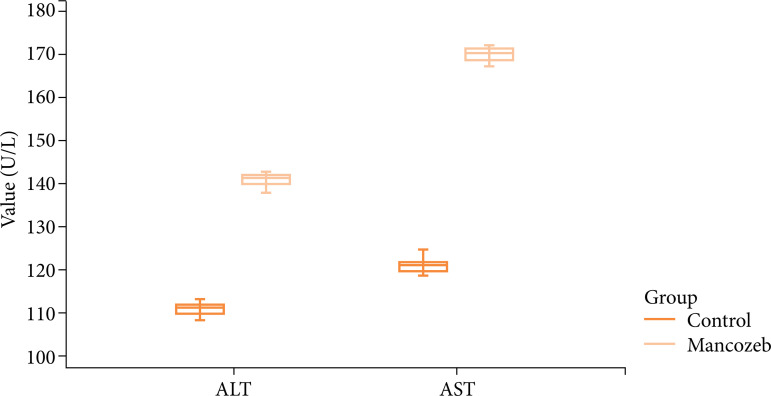
Boxplot graph of AST and ALT values in the control and the mancozeb groups.

AST: aspartate aminotransferase; ALT: alanine aminotransferase.

ALT and AST values of the control and mancozeb groups are shown with boxplot graphics in Fig. 1. Both ALT and AST values of the mancozeb group are significantly higher than the control ones.

## Histopathological results

**Figure 2:**
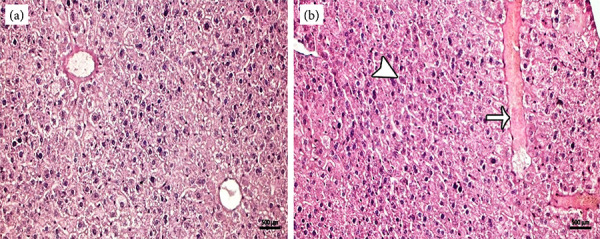
**(a)** Regular vena centralis and hepatocyte cells with a radial appearance in the control group, hematoxylin-eosin staining. **(b)** Dilatation in sinusoidal structures (thin arrow), degeneration in hepatocyte cells(thick arrow) in the mancozeb treated group, hematoxylin-eosin staining.

In the cross-section of the control group, the central vein was regular in the center of the liver lobule, and liver hepatocytes were arranged radially around the nucleus (Fig. 2a). The histopathological section of the liver of the mancozeb-treated group showed sinusoidal dilatation, focal necrosis, significant obstruction, and hemorrhage caused by liver parenchymal changes, as well as significant hepatocellular damage caused by hydropic degeneration and central vascular occlusion (Fig. 2b).

## Immunohistochemistry results

**Figure 3:**
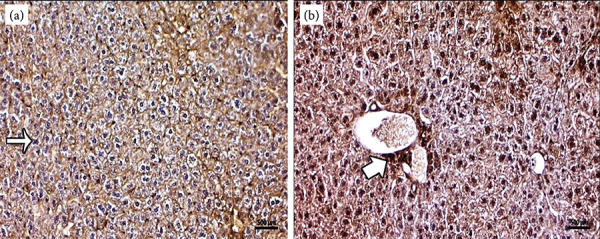
**(a)** Negative caspase-3 expression in hepatocyte and Kupffer cells (arrow) in the control group, caspase-3 immune staining. **(b)** An increase in caspase-3 expression in hepatocyte cells around the vena centralis (thick arrow) in the mancozeb treated group, caspase-3 immune staining.

In the control group sections, caspase-3 immune activity, negative caspase-3 expression was observed in liver cell nuclei and Kupffer cells. In periportal and portal intervals, some connective tissue cells were found to have positive caspase-3 expression (Fig. 3a). In the mancozeb group, at the center of the liver lobule, intensive caspase-3 expression was observed in hepatocyte cells around the central vein (Fig. 3b).

**Figure 4:**
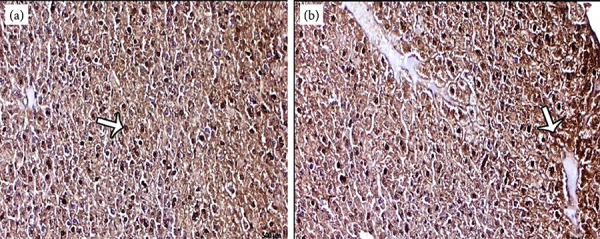
**(a)** Positive TNF-α reaction in some hepatocyte and Kupffer cells (arrow) in the control group, TNF-α immune staining. **(b)** Significant increase in TNF-α expression in hepatocyte and Kupffer cells around vena centralis (arrow) in the mancozeb treated group, TNF-α immune staining.

In the control group, in some of the liver hepatocyte cells and Kupffer cells, TNF-α positive expression was observed (Fig. 4a). In inflammatory cells around the enlarged central vein and Kupffer cells and apoptotic hepatocyte cells of the mancozeb group, increased TNF-α expression was observed (Fig. 4b).

TNF-α: tumor necrosis factor-alpha.

## Discussion

The current study showed that mancozeb has hepatotoxic effects by histopathological, biochemical, and immunohistochemical analysis.

Mancozeb was previously classified as a product with low-acute risk toxicity in humans and with no hepatotoxic effect on rats^29^^,^ 30. However, in many studies conducted on humans and animals, it has been shown that mancozeb causes adverse health effects^31^. Kistinger and Hardej^32^ reported that mancozeb, a fungal form of ethylene bisdithiocarbamate, caused copper accumulation in the kidneys, but no accumulation in the liver. According to the study data, they stated that the ethylene bisdithiocarbamate backbone of mankozeb, not the zinc or manganese moieties, was responsible for the changes in glutathione status and essential metal homeostasis in rat liver and kidney.

Similarly, the efficacy of curcumin in reducing mancozeb-induced hepatotoxicity and genotoxicity in rats was investigated in a experimental study. In conclusion, concomitant treatment with curcumin and mancozeb appeared to minimize the increased levels of liver function markers in serum, lipid peroxidation, proinflammatory mediators and DNA damage parameters in the liver^33^. They aimed to determine the amelioration potential of *Nigella sativa* oil against hepatotoxicity induced by carbendazim and/or mancozeb in female rats. Their results showed that pretreatment with *Nigella sativa* oil significantly reduced carbendazim and mancozeb-induced macrocytic hypochromic anemia, leukocytosis, lymphocytosis, eosinophilia, and neutropenia. It also minimized the incidence of elevated liver enzymes, lipid peroxidation, micronucleus incidence, DNA damage and chromosomal aberrations^34^. It was suggested that daily fungicide-mancozeb exposure in rabbits resulted in maternal mortality, spontaneous abortion, thyroid effects, maternal body weight gain decrements, and decreased bodyweight^35^. In rats and rabbits exposed to a mancozeb-ETU metabolite, noticeable fetal malformations have been verified, including hydrocephaly and domed head (mancozeb). Disruptive, teratogenic, mutagenic, carcinogenic, and endocrine risks are associated with the longer-term toxicity of mancozeb^7^^,^ 9. Genotoxic and pre-malignant changes in human ovarian and immune cells following mancozeb exposure have been shown in recent studies. In humans exposed to mancozeb, increased potential for cancer, and reproductive health risks have also been confirmed by researchers^2^^,^ 36.

Yahia et al.^37^ used 250-mg/kg mancozeb dissolved in corn oil administered twice weekly for seven weeks and indicated that mancozeb exposure caused an increment in triglycerides, and total cholesterol also decreased glucose levels, resulting in the damage of DNA in the liver and colon with pathological changes in stomach, colon, and liver. In the light of this study, we used a single dosage of 500-mg/kg mancozeb dissolved in corn oil. In the current study, the changes in ALT and AST activity among liver damage markers caused lipid-induced oxidative damage and increased cellular degeneration. In the histopathological examination of the liver in mancozeb application, it was found to cause significant hepatocellular damage due to sinusoidal dilatation, focal necrosis, significant obstruction and bleeding, liver parenchymal, as well as hydropic degeneration, and central vascular occlusion.

A study was conducted on rat thymocytes treated with mancozeb (0.01 mg/mL) for a 24-h incubation examined levels of cell viability, apoptosis, mitochondrial membrane potential (MMP), Bcl-2, Bax protein expression, caspase-3, and -9 activity, and p38 mitogen-activated protein kinase (MAPK) signaling involvement. Increased cell toxicity, hypodiploid cells, caspase-3, and -9 activity, Bax protein expression, followed by decreased MMP and Bcl-2 protein expression, were found in cells treated with mancozeb. In thymocytes exposed to mancozeb, apoptosis rate, and caspase-3 activity were significantly reduced through the inhibition of the p38 MAPK signaling pathway^35^. In our study, caspase-3 activity was found to be increased in hepatocyte cells around the central vein in the liver lobule and connective tissue cells in the periportal area in the mancozeb treated group. It was observed that apoptotic change started from the center of the liver lobule to the periphery, and the degenerative cell density was evident around the vessel.

Since mancozeb leads to a significant number of toxic effects on hepatic cell metabolism, another study was conducted with mancozeb on the human HepG2 cell model. It was found that mancozeb caused nonalcoholic fatty liver disease in the human HepG2 cell model, and the 3-(4,5-di-2-yl)-2,5-ditetrazolium bromide (MTT) dye test was used to evaluate total cell death. It was shown that fatty acid-induced steatosis was stimulated via an increased amount of intracellular lipid droplets. Thus, it was found that mancozeb changed cell metabolism and caused cell death through the upregulation of lactate dehydrogenase and cytochrome C^38^.

A study on the consumption of mancozeb-treated vegetables and their effects on rat livers verified that mancozeb contributes to the occurrence of liver disease^39^. Ksheerasagar and Kaliwal^40^ observed that the weight of liver and protein quantity were decreased when mice were exposed daily to mancozeb for 30 days. In the presence of some natural substances (urea, glycine, oxalic acid, and imidazoline), mancozeb degrades^3^. In the case of reacting with mancozeb, these molecules affect blood soluble enzymes and indicators of stress conditions, leading to a situation to change several cells pathways^37^. Thus, an increase occurs in inflammatory parameters that change protein and fat metabolism, including urea, creatinine, hypoalbuminemia, electrolyte disturbances, and some liver enzymes^41^.

TNF-α is a major proinflammatory cytokine with a wide range of studies. TNF-α has been reported to play a role as a cytokine, contributing to the formation and development of liver damage^18^. Proinflammatory cytokines such as TNF-α, IL6, IL-1, and chemokines have been reported to be produced and released by Kupffer cells together with adjacent non-parenchymal cells as a result of damage and necrosis^42^. In this study, TNF-α activity of mancozeb-induced liver injury of TNF-α was evaluated. Due to increased apoptosis, increased inflammation in the liver has led to an increase in TNF-α level.

## Conclusions

The subacute administration of mancozeb in rats with impaired liver function increased inflammation in tissue, and increased apoptosis due to cellular damage may lead to elevated toxicity in the liver and decreased liver regeneration ability due to congestion and degeneration of blood vessels. As a pesticide, especially unconsciously, high doses and subacute use of mancozeb can cause serious liver tissue damage. Physicians should be aware of the toxicity of mancozeb in such toxication cases.
